# Post-systolic shortening is superior to global longitudinal strain in predicting adverse events in patients with stable coronary artery disease and preserved systolic function

**DOI:** 10.1186/s13244-022-01174-y

**Published:** 2022-03-02

**Authors:** Shirui Lu, Xin Hu, Jun Zhang, Ying Zhu, Wei Zhou, Yani Liu, Youbin Deng

**Affiliations:** Department of Medical Ultrasound, Tongji Hospital, Tongji Medical College, Huazhong University of Science and Technology, 1095 Jiefang Road, Wuhan, 430030 China

**Keywords:** Stable coronary artery disease, Speckle tracking echocardiography, Automated functional imaging, Post-systolic shortening, Global longitudinal strain

## Abstract

**Background:**

Post-systolic shortening (PSS) is an important indicator for early identifying myocardial dysfunction. We aimed to investigate the predictive value of PSS assessed with speckle tracking automated functional imaging (AFI) on adverse events in patients with stable coronary artery disease (CAD) and preserved systolic function.

**Methods:**

A total of 204 consecutive patients clinically diagnosed with stable CAD and left ventricular ejection fraction (LVEF) > 50% were included. Multiple parameters were analyzed with AFI technique. The composite endpoint included all-cause mortality, heart failure, myocardial infarction and stroke.

**Results:**

During a median follow-up of 24 months (IQR 19–28 months), 30 patients (14.7%) reached the endpoint. Patients experiencing the endpoint had a lower absolute global longitudinal strain (GLS), a higher post-systolic index (PSI), and more left ventricle walls displaying PSS than patients without events. PSI (hazard ratio, 1.15; 95% CI, 1.04–1.27; *p* = 0.005) and per 1 increase in the number of left ventricle walls with PSS (hazard ratio 1.52, 95% CI 1.21–1.91, *p* < 0.000) were independent predictors of the endpoint, whereas GLS was not significantly associated with the endpoint after adjustment models. For patients with absolute value of GLS > 15.4%, a significant prognostic superiority was found in PSI compared with GLS (AUC = 0.73 [PSI] vs. 0.58 [GLS], *p* = 0.024).

**Conclusions:**

PSS is an independent predictor for adverse events in stable CAD patients with preserved systolic function, and the prognostic value may be superior to GLS in patients with normal or mildly reduced GLS.

**Supplementary Information:**

The online version contains supplementary material available at 10.1186/s13244-022-01174-y.

## Key points

- GLS and PSS can early identify myocardial dysfunction.
- PSI and LV walls with PSS can independently predict adverse outcomes.
- PSS may have a superiority predicting adverse outcomes when GLS was preserved.

## Introduction

Coronary artery disease (CAD) remains the leading cause of death in developed and developing countries [1]. Stable CAD refers to known CAD with no recent or acute events, encompassing a diverse spectrum of patients. Notably, the concept of 'stable' CAD is misleading due to the continuing risks of cardiovascular events over the longer term and the heterogeneous risk characteristics [2]. It has been reported that the probability of having major adverse cardiovascular events (MACEs) within five years of the onset of apparently stable angina is up to 35%, depending on clinical variables that affect the risk [3]. Therefore, it is of great value to improve the abilities during the work-up in identifying those patients with increased risk of adverse outcomes.

Transthoracic echocardiography is the commonly used non-invasive imaging method for the evaluation of CAD, which was shown not only to aid diagnosis but also to provide valuable prognostic information in the clinical setting [4]. Compared to the traditional left ventricular ejection fraction (LVEF), a growing literature found that speckle tracking echocardiography (STE) could provide more sensitive parameters in early identifying subtle myocardial damage, particularly contributing to improving the prognostic assessment in the patients with preserved LVEF [5]. In a study of 659 patients after acute myocardial infarction, global longitudinal strain (GLS) was independently related to all-cause mortality, reinfarction, revascularization, and heart failure hospitalization at 3-year follow-up and was found to be superior to LVEF and wall motion score index (WMSI) [6].

In addition to longitudinal strain, post-systolic shortening (PSS) has recently attracted attention as another marker of myocardial ischemia [7]. PSS is defined as a delayed systolic shortening occurring after the aortic valve closure. The most commonly used parameter to quantify PSS is the post-systolic index (PSI) which is calculated as [8]:

1 $${\text{PSI}} = \frac{{{\text{maximal strain }} - {\text{ peak systolic strain}}}}{{\text{maximal strain}}} \times 100$$

where maximal strain and peak systolic strain indicate the peak negative strain in cardiac cycle and in systole, respectively.

It is reported that PSS may occur in passive dyskinesia and also at a low magnitude in healthy individuals [9]. According to Voigt et al. [10], pathological PSS is defined as at least one segment of left ventricle (LV) myocardium PSI (Eq. 1) > 20%. The superiority of PSS to conventional parameters in detecting acute ischemia and predicting adverse cardiovascular events has been indicated in clinical studies [11, 12]. Interestingly, among the myocardial deformation parameters derived from STE, PSS is sometimes observed without a relative strain decrease in the acute ischemic region, especially after recovery from ischemia [13]. In a recent animal study, PSS remained in the risk area at 20 min after reperfusion significantly, although peak systolic strain recovered by 20 min [14]. Currently, some authors consider the assessment of PSS during STE analysis in patients with CAD as equally or more critical to commonly used systolic strain, particularly in a state of acute ischemia or systolic stunning [7, 15]. However, there is still a lack of data concerning PSS and its prognostic significance compared with LVEF and systolic strain in stable CAD population.

Different from the traditional manually tracing the myocardium, a novel algorithm of STE, named automatic functional imaging (AFI), can automatically and quickly provide multiple strain parameters, in turn improving workflow and reducing user variability [16]. Therefore, AFI algorithm was used in the present study to explore the prognostic value of AFI multiple-parameter analysis in stable CAD patients with preserved ejection fraction, which would be more challenging in clinical setting. Moreover, whether PSS assessed by AFI in stable CAD patients could provide powerful prognostic information on the adverse cardiovascular events and its superiority to GLS was fully explored.

## Methods

### Study population

We prospectively studied 297 consecutive patients with clinically diagnosed stable CAD from April 2016 to December 2020. Inclusion criteria were (1) suspected CAD patients had stable intermittent chest pain or exertional dyspnea related to myocardial ischemia according to a comprehensive clinical investigation including location, character, duration and relationship to exertion and other exacerbating or relieving factors. (2) No changes in frequency, duration, precipitating causes or relief for at least two months before admission. All patients underwent transthoracic echocardiography combined with speckle tracking AFI analysis. Critical exclusion criteria were (1) coronary angiography revealed < 50% reduction of the arterial lumen area in any major coronary artery; (2) LVEF ≤ 50%; (3) a history of significant heart failure, acute coronary syndrome within the last year; (4) other heart diseases including cardiomyopathy and significant valvular heart disease, ventricular arrhythmia, atrial fibrillation, left or right bundle branch block or paced rhythm, and other serious non-cardiac diseases; (5) suboptimal image quality and arrhythmia that may influence the analysis of speckle tracking AFI. A total of 204 patients were finally included in this study. Tongji Hospital Ethics Committee approved the study, and informed consent was obtained from all patients before the study.

### Transthoracic echocardiography

Transthoracic echocardiograms were performed using GE Vivid E95 ultrasound equipment (GE Vingmed Ultrasound, Horten, Norway) with an M5Sc transducer (1.7–3.3 MHz) and a high frame rate (70–80 frame/s). LV parasternal long-axis view, short-axis views at basal, midventricular and apical levels, as well as apical 2-, 3- and 4-chamber views were acquired. All echocardiograms were analyzed in accordance with the quantitative method recommended by the American Society of Echocardiography [17]. The interventricular septum (IVS) thickness, left ventricular posterior wall (LVPW) thickness and left ventricular end-diastolic dimension (LVEDD) were measured in the parasternal long-axis view at the level of mitral valve leaflet tips. LV mass index (LVMI) was calculated as the anatomic mass divided by the body surface area. Biplane Simpson’s method was used to determine LV end-systolic volumes (LVESV), LV end-diastolic volumes (LVEDV) and LVEF. In the four-chamber apical view, pulsed-wave Doppler was used to assess mitral valve inflow velocities, including peak velocity of early (E) and late (A) diastolic LV filling. E/A ratio was calculated. Pulsed-wave tissue Doppler was also used to measure peak early diastolic (e′) velocities at the septal mitral annular in the four-chamber view. The E/e′ was calculated. All echocardiograms were analyzed by an investigator who was blinded to all clinical baseline and outcome data.

### Speckle tracking automated functional imaging

**Fig. 1:**
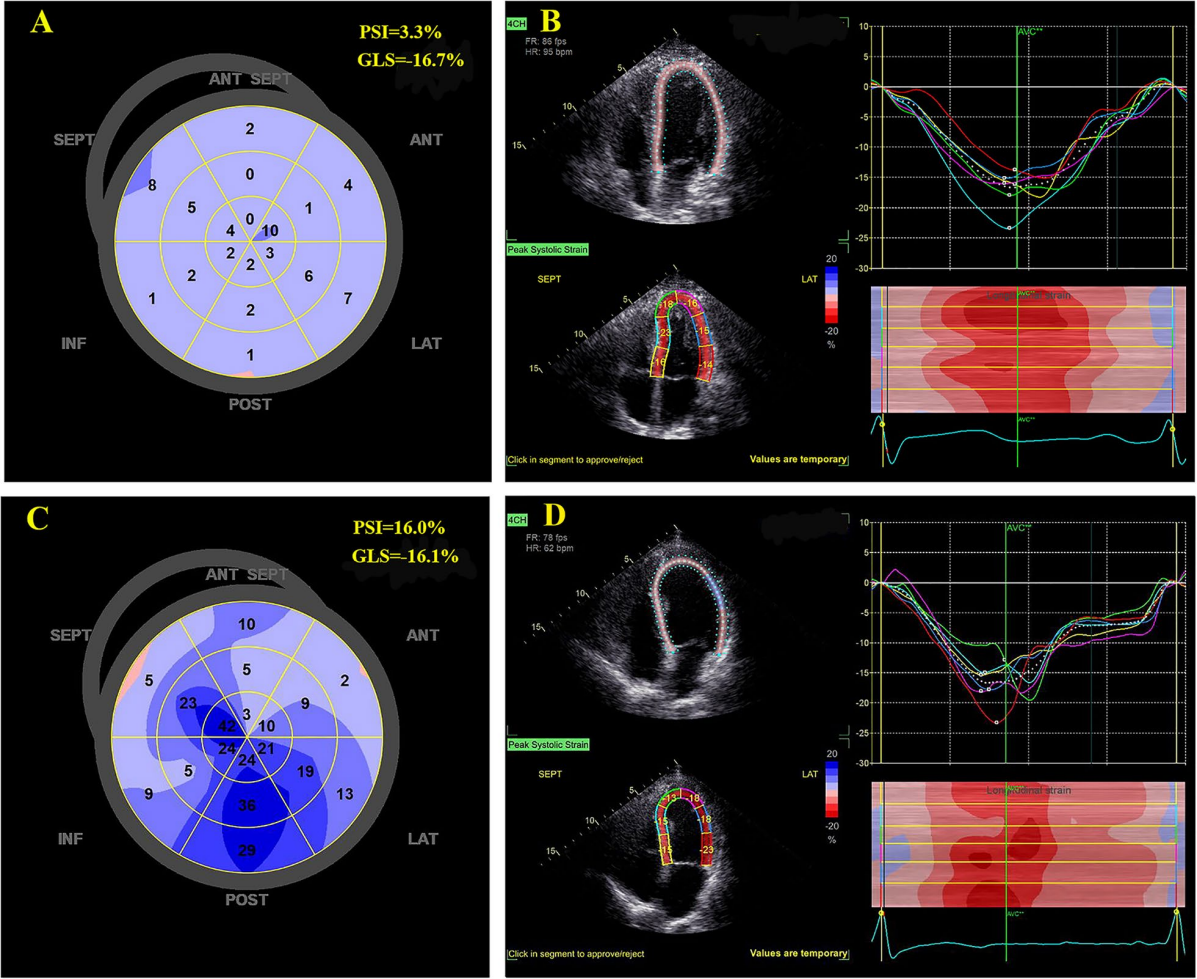
Representative cases. The bull’s eye map of post-systolic index (PSI) and longitudinal strain curves of six myocardial segments in the apical four-chamber view of the patients without (**A**, **B**) and with (**C**, **D**) major adverse cardiovascular events during follow-up. Both patients showed normal global longitudinal strain (GLS) and left ventricular ejection fraction (LVEF). **A** Normal bull’s eye map of PSI with an average value of 3.3%. **B** Normal strain curves with little or no sign of post-systolic shortening (PSS). **C** Abnormal bull’s eye map of PSI with an increased average value of 16.0%. **D** Abnormal strain curves where the green segment exhibits obvious PSS

The commercial speckle tracking AFI software (version 2.3, GE Vingmed Ultrasound) was used to perform two-dimensional (2D) strain analysis in apical two-, three- and four-chamber views. The AFI software analyses myocardial motion by tracking frame-to-frame speckle changes in 2D images [18]. When necessary, automatic endocardial recognition was manually adjusted to ensure correct ‘anchorage’ of the algorithm to the mitral annulus, exclude papillary muscles and chordae from tracking and correctly include the LV apex. The region of interest (ROI) was eventually adjusted to ensure tracking of the whole myocardial thickness [19]. LV outflow pulsed Doppler was used to time end systole [20]. The segmental strain curves in apical view and 18-segment bull's-eye diagrams related to strain parameters were automatically displayed (Fig. 1).

GLS was calculated as the average value of the peak systolic strain in LV 18 myocardial segments [21]. PSI of each segment was calculated as Eq. (1) (Additional file 1: Fig. S1). When post-systolic shortening was not observed, PSI was set to zero. Overall PSI was obtained as the average from the 18 myocardial segments. In the categorical analysis, the presence of PSS was defined as PSI > 20% in at least one myocardial segment. This cutoff value was based on previous evidence evaluating PSS [10]. If a segment within a myocardial wall displayed PSS, the wall was categorized as having the presence of PSS.

### Coronary angiography

All patients underwent coronary angiography to confirm the diagnosis with CAD. Coronary angiography was performed by two experienced interventionists using standard technique [22]. Stenosis with ≥ 50% reduction of the arterial lumen area in at least one major coronary artery (including left main artery [LM], the left anterior descending artery [LAD], left circumflex artery [LCX] and right coronary artery [RCA]) was considered CAD.

### Follow-up

All participants were followed from July 2016 to August 2021 or the time of the event. The composite endpoint included all-cause mortality, heart failure, myocardial infarction and stroke. If a patient experienced several events, the first occurring event was used in the composite end point. The end points were obtained from our hospital’s Patient Registry and telephone calls. Fifteen patients lost to follow-up were excluded in the final analysis.

### Reproducibility

To define intra- and inter-observer variability, 30 patients were randomly selected and remeasured by two observers blinded to patient data and each other's results. One of the researchers did the analysis once again after two weeks. Intra- and inter-observer reproducibility and variability were calculated by intraclass correlation coefficient (ICC).

### Statistical analysis

All analyses were performed using SPSS version 21.0 statistical analysis software (IBM, Armonk, NY, USA), MedCalc version 15.2.2 (MedCalc Software, Mariakerke, Belgium) and R version 4.1.1 (R Foundation for Statistical Computing, Vienna, Austria). Normally distributed continuous data are presented as mean ± standard deviation. Non-normally distributed continuous data are presented as median and interquartile range (IQR). Shapiro–Wilk test and q-q plots were used to check continuous values for normality of the data distribution. The differences between two groups were assessed using Student’s t test for normally distributed data and Mann– Whitney U test for non-normally distributed data. The differences among the three groups were analyzed using the one-way analysis of variation (ANOVA) test for normally distributed data with the Bonferroni correction for pairwise comparisons between the data in each group. The Kruskal–Wallis rank-sum test was used for non-normally distributed data in three groups, and the all pairwise method was used for further pairwise comparisons between two groups. Categorical data are presented as percentages and statistically analyzed with the χ^2^ test or Fisher's exact test (as appropriate).

Receiver operator characteristic (ROC) curve analysis was performed to investigate the predictive ability of different indicators for adverse events. From the analysis of the ROC, areas under the curve (AUC) and 95% confidence intervals were obtained. By maximization of Youden's index, the optimal cutoff values with specificity or sensitivity were calculated. Comparison of AUC was performed using the method of DeLong in MedCalc. This cutoff value was used to construct Kaplan–Meier survival curves, and associations between the respective echocardiographic parameters and endpoints were compared by the log-rank test. Cox proportional hazards method was used to calculate hazard ratios (HRs) and 95% confidence intervals (CIs). For multivariable COX proportional hazards regression analyses, LVEF and variables with significant P value < 0.05 in univariable analyses were included in the multivariable models to detect independent risk factors for predicting adverse events. Restricted cubic spline model was constructed to analyze the relationship between PSI and the hazard ratio of adverse events. A two-way random-effects model was used with absolute agreement-type analysis for intraclass correlation coefficient (ICC). A two-tailed *p* value < 0.05 was considered a statistically significant difference.

## Results

### Baseline patient characteristics

A total of 297 participants diagnosed with stable CAD were examined by conventional echocardiography and STE. Of these participants, 78 were excluded because of exclusion criteria and 15 patients lost to follow-up. A total of 204 patients (mean age: 59 ± 10 years, 73% men) were finally evaluated in this study (Additional file 1: Fig. S2). During a median follow-up of 24 months (IQR 19–28 months), 30 patients (14.7%) reached the composite endpoint, including 5 patients underwent heart failure, 13 patients experienced myocardial infarction, 4 patients admitted for stroke, and 8 patients died. The cause of death was cardiac in 4 patients, noncardiac in 1 patient and unknown in the remaining 3 patients.

Patient characteristics stratified by follow-up outcome are summarized in Table 1. Patients with events were generally older, showing a higher proportion of New York Heart Association (NYHA) functional class III-IV, a lower absolute GLS − −15.2 ± 3.1% vs. − 17.4 ± 2.8%, *p* < 0.05), a higher PSI (12.8% [IQR, 7.4–15.0%] vs. 6.3% [IQR, 4.4–9.3%], *p* < 0.05) and more LV walls with PSS than patients without events. No significant differences were found regarding E/A, E/e′, ventricular wall thickness, left ventricular volumes and LVEF between the two groups (*p* > 0.05).

**Table 1:** Baseline characteristics stratified by follow-up outcome

	Overall population (*n* = 204)	With event (*n* = 30)	Without events (*n* = 174)	*p* value
*Baseline clinical*				
Age, year	59 ± 10	64 ± 10	58 ± 10	0.009*
Male	148 (73)	21 (70)	127 (73)	0.735
Body mass index, kg/m^2^	22.9 ± 2.7	22.8 ± 2.9	23.0 ± 2.6	0.814
Systolic blood pressure, mm Hg	129 ± 17	134 ± 18	128 ± 17	0.096
Diastolic blood pressure, mm Hg	78 ± 11	79 ± 14	77 ± 11	0.551
Heart rate, bpm	70 ± 10	70 ± 11	71 ± 11	0.729
Smoke	50 (24.5)	7 (23.3)	43 (24.7)	0.871
Hypertension	119 (58.3)	22 (73.3)	97 (55.7)	0.071
Diabetes	72 (35.3)	15 (50.0)	57 (32.8)	0.068
ACEI/AT2-antagonists	81 (39.7)	16 (53.3)	65 (37.4)	0.099
Beta-blockers	121 (59.3)	20 (66.7)	101 (58.0)	0.375
Calcium blocker	99 (48.5)	12 (40.0)	87 (50.0)	0.311
Aspirin	168 (82.4)	25 (83.3)	143 (82.2)	0.879
*Revascularization therapy*				
PCI	33 (16.2)	7 (23.3)	26 (14.9)	0.269
CABG	90 (44.1)	15 (50.0)	75 (43.1)	0.482
Carotid plaque	128 (62.7)	20 (66.7)	108 (62.1)	0.630
ST depression ≥ 0.5 mm	77 (37.7)	13 (43.3)	64 (36.8)	0.494
T wave inversion ≥ 1 mm	67 (32.8)	14 (46.7)	53 (30.5)	0.081
Pathological Q wave ≥ 0.04 s	32 (15.7)	4 (13.3)	28 (16.1)	0.701
*Location of significant stenosis*				
LAD	152 (74.5)	26 (86.7)	126 (72.4)	0.098
LCX	114 (55.9)	21 (70.0)	93 (53.4)	0.092
RCA	111 (54.4)	21 (70.0)	90 (51.7)	0.063
NYHA functional class III–IV	61 (29.9)	16 (53.3)	45 (25.9)	0.002*

Data are presented as mean ± SD or median (interquartile range) for continuous variables, and count (%) for categorical variables

ACEI: angiotensin-converting enzyme inhibitors; AT: angiotensin; PCI: percutaneous coronary intervention; CABG: coronary artery bypass grafting; LAD: left anterior descending artery; LCX: left circumflex artery; RCA: right coronary artery; NYHA: New York Heart Association; IVS: interventricular septum; LVPW: left ventricular posterior ventricular wall; LVEDD: left ventricular end-diastolic diameter; LVEDV: left ventricular end-diastolic volume; LVESV: left ventricular end-systolic volume; LVMI: left ventricular mass index; LVEF: left ventricular ejection fraction; GLS: global longitudinal strain; PSI: post-systolic index; PSS: post-systolic shortening

P-value marks trend for events group vs. without events group (*p < 0.05)

In total, there are 99 (48.5%) patients with no PSS, 62 (30.4%) patients with 1–2 walls of PSS and 43 (21.1%) patients with ≥ 3 walls of PSS. As shown in Table 2, patients with ≥ 3 walls of PSS had a higher proportion of NYHA functional class III-IV and a lower E/A ratio (0.7 [IQR, 0.6–0.9] vs. 0.8 [IQR, 0.7–0.9], *p* < 0.05) than patients with no PSS. Increasing numbers of LV walls with PSS were related to a higher incidence of adverse events, a lower LVEF, a higher LVESV and a lower GLS, such that participants with no PSS had a mean GLS of − 17.7 ± 2.4%, those with 1–2 wall of PSS had a mean GLS of − 17.4 ± 2.8% and those with ≥ 3 walls of PSS had a mean GLS of −15.2 ± 3.5% (*p* trend < 0.05). To some extent, these results indicated that patients with ≥ 3 walls of PSS had impaired LV function compared with patients with < 3 walls of PSS. No significant differences were found regarding left ventricular wall thickness, E/e′ across groups.

**Table 2:** Baseline characteristics stratified by left ventricle walls with pathological PSS

Walls with presence of PSS	No walls (*n* = 99)	1–2 walls (*n* = 62)	≥ 3 walls (*n* = 43)	*p* value
Age, year	58 ± 10	59 ± 10	62 ± 11	0.222
Male	72 (73)	44 (71)	32 (74)	0.925
Body mass index, kg/m^2^	22.7 ± 2.5	23.3 ± 3.0	23.1 ± 2.7	0.353
Systolic blood pressure, mm Hg	128 ± 18	129 ± 17	130 ± 16	0.795
Diastolic blood pressure, mm Hg	78 ± 11	77 ± 10	78 ± 12	0.749
Heart rate, bpm	71 ± 10	70 ± 9	70 ± 10	0.797
Smoke	29 (29.3)	10 (16.1)	11 (25.6)	0.165
Hypertension	55 (55.6)	35 (56.5)	29 (67.4)	0.392
Diabetes	36 (36.4)	16 (25.8)	20 (46.5)	0.088
Carotid plaque	60 (60.6)	38 (61.3)	30 (69.8)	0.561
ST depression ≥ 0.5 mm	38 (39.4)	21 (32.3)	18 (41.9)	0.697
T wave inversion ≥ 1 mm	40 (40.4)	20 (32.3)	18 (41.9)	0.503
Pathological Q wave ≥ 0.04 s	15 (15.2)	8 (12.9)	9 (20.9)	0.528
*Location of significant stenosis*				
LAD	70 (71.7)	47 (75.8)	35 (81.4)	0.390
LCX	52 (52.5)	34 (54.8)	29 (67.4)	0.247
RCA	48 (48.5)	35 (56.5)	28 (65.1)	0.174
NYHA functional class III–IV	20 (20.2)	23 (37.1)	18 (47.9)^#^	0.012*
*Echocardiography*				
IVS, mm	10 ± 1	10 ± 1	11 ± 1	0.065
LVPW, mm	10 ± 3	10 ± 1	10 ± 1	0.933
LVEDD, mm	45 ± 6	45 ± 5	46 ± 4	0.385
LVEDV, mL	88 ± 22	86 ± 22	95 ± 27	0.154
LVESV, mL	35 ± 11	35 ± 10	41 ± 13^#&^	0.008*
LVMI, g/m^2^	87.6 (75.6, 100.0)	89.9 (77.0, 109.7)	95.9 (79.2, 118.1)	0.060
E/A	0.8 (0.7, 0.9)	0.8 (0.6, 1.0)	0.7 (0.6, 0.9)^#^	0.040*

Data are presented as mean ± SD or median (interquartile range) for continuous variables, and count (%) for categorical variables

LAD: left anterior descending artery; LCX: left circumflex artery; RCA: right coronary artery; NYHA: New York Heart Association; IVS: interventricular septum; LVPW: left posterior ventricular wall; LVEDD: left ventricular end-diastolic diameter; LVEDV: left ventricular end-diastolic volume; LVESV: left ventricular end-systolic volume; LVMI: left ventricular mass index; E/A: mitral inflow peak early velocity/mitral inflow peak late velocity; E/e′: mitral inflow peak early velocity/mitral annular peak early velocity; LVEF: left ventricular ejection fraction; GLS: global longitudinal strain

^#^*p* < 0.05 versus no walls group. ^&^*p* < 0.05 versus 1–2 walls group

### Event risk prediction by conventional echocardiographic and AFI parameters in stable CAD

ROC curves analyses of multiple parameters to predict adverse events in stable CAD are illustrated in Table 3. Among these echocardiography parameters, PSI (AUC = 0.76 [PSI] vs. 0.55 [LVEF], *p* = 0.003; 0.76 [PSI] vs. 0.54 [E/A], *p* = 0.002; 0.76 [PSI] vs. 0.53 [E/e′], *p* = 0.002) was superior to conventional parameters in predicting adverse events. The cutoff value was 10.4% for PSI with a sensitivity of 63.3% and specificity of 81.0%. Similarly, GLS (AUC = 0.70 [GLS] vs. 0.55 [LVEF], *p* = 0.040; 0.70 [GLS] vs. 0.54 [E/A], *p* = 0.027; 0.70 [GLS] vs. 0.53 [E/e′], *p* = 0.049) was also superior to conventional parameters in predicting adverse events. The cutoff value was −15.4% for GLS with a sensitivity of 56.7% and specificity of 77.0%, whereas no significant difference was found between PSI and GLS (AUC = 0.76 [PSI] vs. 0.70 [GLS], *p* = 0.249) (Additional file 1: Fig. S3).

**Table 3:** Receiver operating characteristic curve analysis of multiple parameters to predict adverse events

	AUC	95% CI	*p* value	*p* value (VS GLS)	*p* value (VS PSI)	Cutoff value	Sensitivity	Specificity
LVEF, %	0.55	0.48–0.62	0.334	0.040*	0.003*	60	70.0	46.6
E/A	0.54	0.47–0.61	0.440	0.027*	0.002*	0.8	56.7	55.2
E/e′	0.53	0.46–0.60	0.648	0.049*	0.002*	12.2	50.0	75.9
GLS, -%	0.70	0.63–0.76	< 0.001*	/	0.249	15.4	56.7	77.0
PSI, %	0.76	0.70–0.82	< 0.001*	0.249	/	10.4	63.3	81.0

AUC: area under the curve; CI: confidence interval; LVEF: left ventricular ejection fraction; E/A: mitral inflow peak early velocity/mitral inflow peak late velocity; E/e′: mitral inflow peak early velocity/mitral annular peak early velocity; GLS: global longitudinal strain; PSI: post-systolic index

*p < 0.05

### Impact of conventional echocardiographic and AFI parameters on the adverse events in stable CAD

**Fig. 2:**
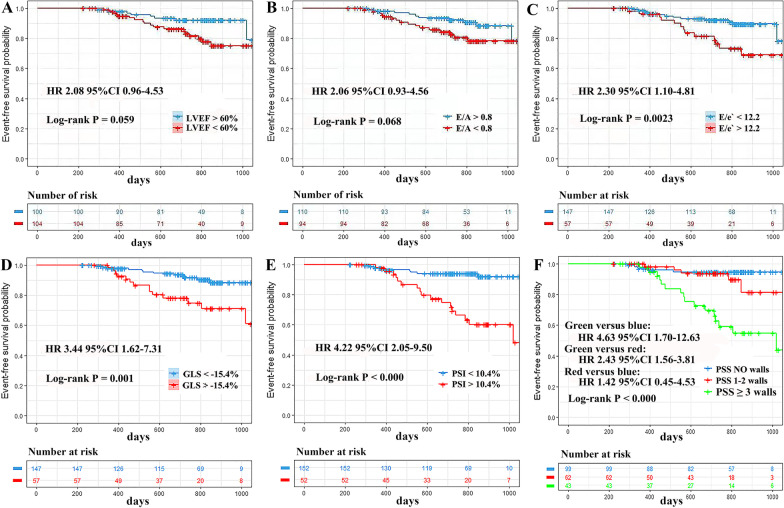
Event-free survival. Kaplan–Meier survival curves for adverse events prediction by (**A**) LVEF; (**B**) E/A; (**C**) E/e′; (**D**) GLS; (**E**) PSI; (**F**) the number of left ventricle walls with PSS. LVEF: left ventricular ejection fraction; E/A: mitral inflow peak early velocity/mitral inflow peak late velocity; E/e′: mitral inflow peak early velocity/mitral annular peak early velocity; GLS: global longitudinal strain; PSI: post-systolic index; PSS: post-systolic shortening

Kaplan–Meier event-free survival curves are shown in Fig. 2. All spackle tracking indices (GLS, PSI and numbers of LV walls with PSS) had significant differences in survival time at different levels (log-rank *p* < 0.05 for all parameters), whereas conventional parameters (LVEF, E/A) except E/e′ showed no significant differences in survival time at different levels (log-rank *p* > 0.05).

In univariable Cox regression analysis (Table 4), age (HR 1.06, 95%CI 1.01–1.10, *p* = 0.008), hypertension (HR 2.38, 95%CI 1.01–5.59, *p* = 0.046), NYHA functional class III-IV (HR 2.24, 95%CI 1.05–4.76, *p* = 0.036), GLS (HR 0.85, 95%CI 0.76–0.95, *p* = 0.004), PSI (HR 1.19, 95%CI 1.09–1.29, *p* = 0.001) were significantly associated with adverse events, while no significant associations were found in LVEF, E/A and E/e' (*p* > 0.05 for all). Per 1 increase in number of LV walls with PSS, the risk of adverse events increased by 1.69 times (95%CI 1.39–2.05, *p* < 0.000). LVEF and all variables with *p* < 0.05 in univariable Cox regression analysis were included in multivariable Cox regression analysis. Both the number of walls with PSS (per 1 increase in the number of walls with PSS: HR 1.52, 95%CI 1.21–1.91, *p* < 0.000) and PSI (HR 1.15, 95%CI 1.04–1.27, *p* = 0.005) remained independent predictors, but no remained significant prediction was observed in GLS after adjustment.

**Table 4:** Cox regression analysis of multiple variables on the composite endpoint

	Univariable analysis		Multivariable analysis			
	HR (95% CI)	*P* value	Model 1 HR (95% CI)	*P* value	Model 2 HR (95% CI)	*P* value
Male	1.06 (0.46–2.40)	0.898				
Age	1.06 (1.01–1.10)	0.008*	1.03 (0.99–1.07)	0.137	1.03 (0.99–1.07)	0.102
Body mass index, kg/m^2^	1.00 (0.87–1.14)	0.952				
Heart rate, bpm	0.99 (0.95–1.03)	0.602				
Systolic blood pressure, mmHg	1.02 (1.00–1.04)	0.060				
Diastolic blood pressure, mmHg	1.03 (1.00–1.06)	0.081				
Revascularization therapy	1.42 (0.59–3.41)	0.429				
Hypertension	2.38 (1.01–5.59)	0.046*	2.12 (0.86–5.25)	0.102	1.71 (0.69–4.28)	0.250
Diabetes	1.65 (0.78–3.45)	0.187				
Carotid plaque	1.22 (0.55–2.68)	0.625				
Number of coronary stenoses	1.18 (0.84–1.66)	0.336				
NYHA functional class III–IV	2.24 (1.05–4.76)	0.036*	1.45 (0.66–3.18)	0.350	1.29 (0.59–2.80)	0.523
LVEF, %	0.94 (0.88–1.01)	0.104	0.97 (0.90–1.05)	0.422	0.97 (0.90–1.05)	0.508
E/A	0.22 (0.04–1.22)	0.082				
E/e′	0.97 (0.87–1.07)	0.560				
GLS, -%	0.85 (0.76–0.95)	0.004*	0.95 (0.84–1.08)	0.419	0.96 (0.85–1.09)	0.965
PSI, %	1.19 (1.09–1.29)	0.001*	1.15 (1.04–1.27)	0.005*		
Per 1 increase in number of walls with PSS	1.69 (1.39–2.05)	< 0.000*			1.52 (1.21–1.91)	< 0.000*

HR: hazard ratio; CI: confidence interval; NYHA: New York Heart Association; LVEF: left ventricular ejection fraction; E/A: mitral inflow peak early velocity/mitral inflow peak late velocity; E/e′: mitral inflow peak early velocity/mitral annular peak early velocity; GLS: global longitudinal strain; PSI: post-systolic index; PSS: post-systolic shortening

*p < 0.05

**Fig. 3:**
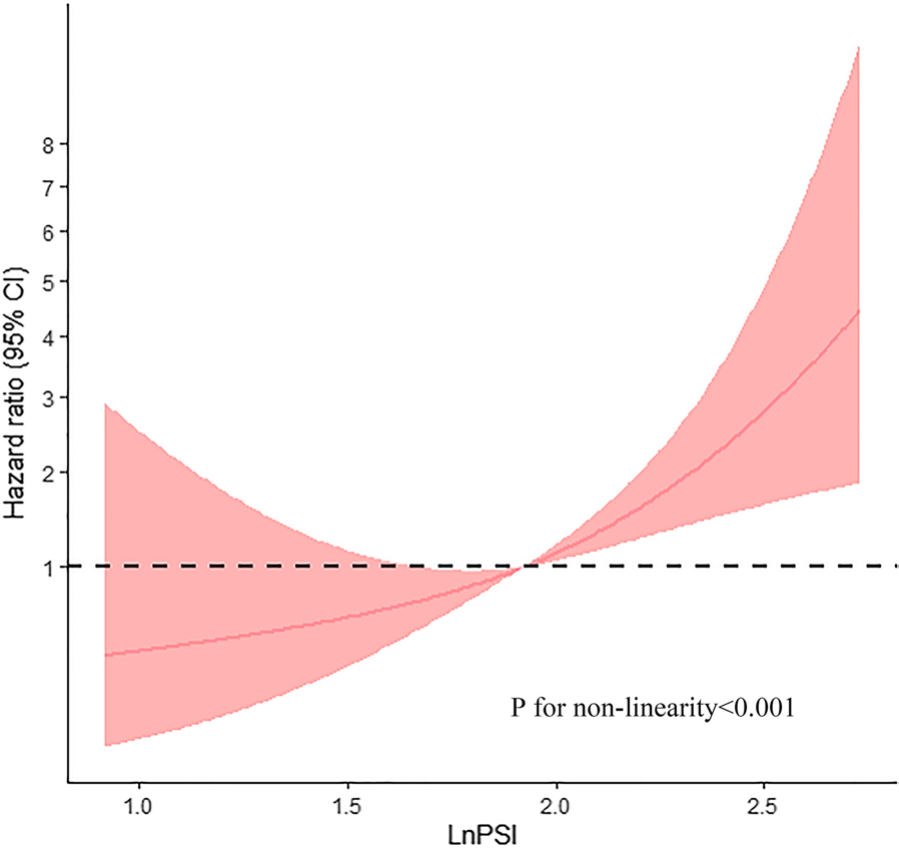
Restricted cubic splines of nonlinear association between LnPSI and hazard ratio. The bold red lines indicate the pooled restricted cubic spline model, and the red filled area indicates the 95% confidence intervals of the pooled curve. PSI: post-systolic index

PSI showed a non-Gaussian distribution, which was successfully converted to a Gaussian distribution using logarithmic transformation. The association between PSI and risk of adverse events was evaluated on a continuous scale with restricted cubic spline curves based on Cox proportional hazards models. The increasing PSI was associated with significantly increased risk of adverse events. Figure 3 shows a significant *p* < 0.001 for nonlinearity PSI–HR.

**Fig. 4:**
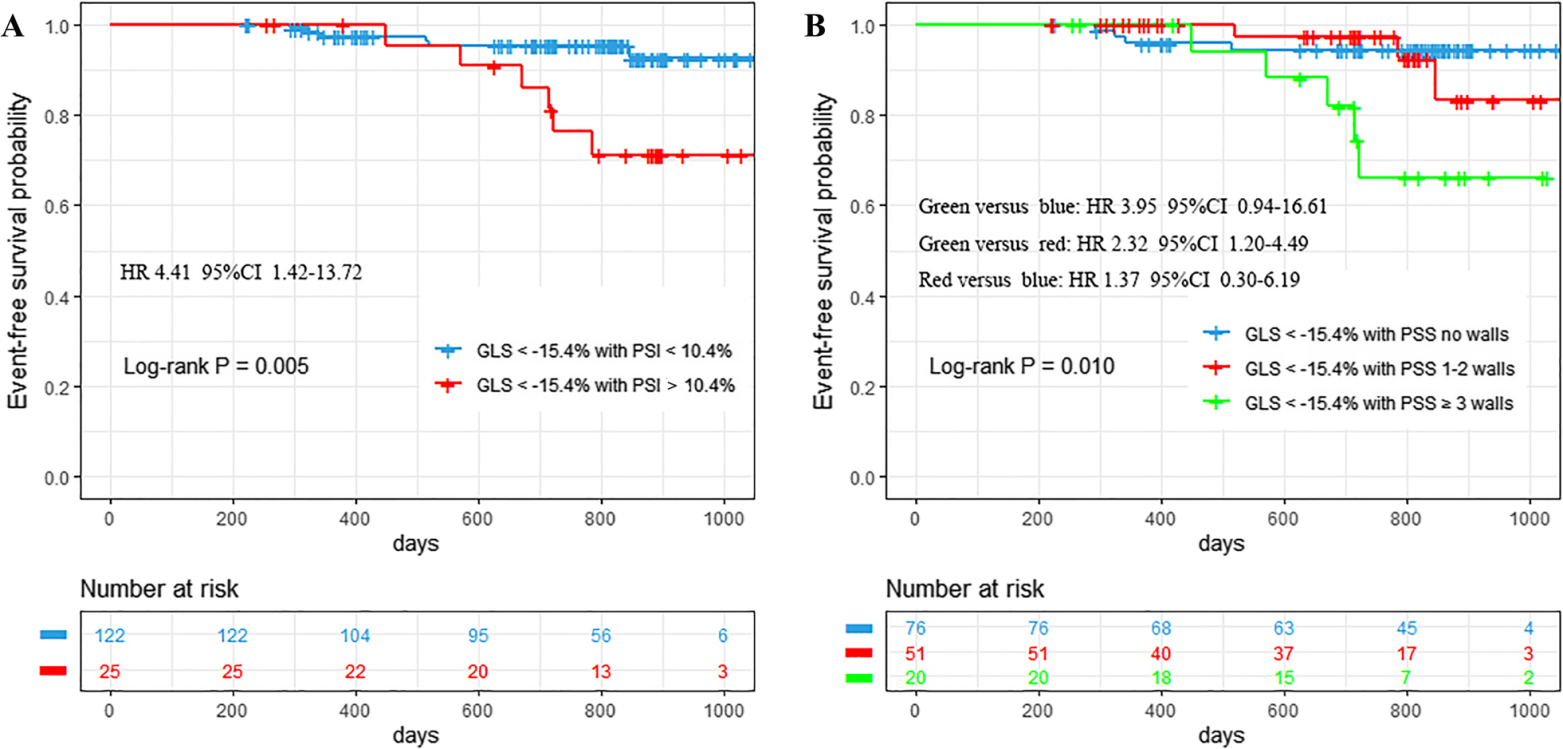
Event-free survival. Kaplan–Meier survival curves for adverse events prediction in patients with stratification of GLS > 15.4% by (**A**) GLS; (**B**) the number of left ventricle walls with PSS. GLS: global longitudinal strain; PSI: post-systolic index; PSS: post-systolic shortening

The prediction performance of PSS was further explored in patients with the absolute value of GLS > 15.4%, representing patients with sub-clinical myocardial dysfunction. In these patients, a significant prognostic superiority was found in PSI compared with GLS (AUC = 0.73[PSI] vs. 0.58[GLS], *p* = 0.024). These results indicated that for patients with normal or mildly reduced GLS, the prognostic value of PSS may be superior to GLS. Furthermore, Kaplan–Meier survival curves showed that patients with both GLS and PSI beyond the prognostic cutoff values had significantly higher adverse events risk than patients with only GLS beyond the prognostic cutoff value (HR 4.41, 95%CI 1.42–13.72, Log-rank *p* = 0.005). For patients with the absolute value of GLS > 15.4%, ≥ 3 walls of PSS showed a higher risk of adverse events than 1–2 walls of PSS (HR 3.95, 95%CI 0.94–16.61) and no walls of PSS (HR 2.32, 95%CI 1.20–4.49) (log-rank *p* = 0.010) (Fig. 4).

### Inter-observer and intraobserver variability analyses

As demonstrated in Additional file 1: Table S1, both intra- and inter-observer intraclass correlation coefficients indicate good or excellent reliability for all strain parameters.

## Discussion

This prospective study explored the prognostic value of myocardial deformation parameters in stable CAD patients with preserved systolic function. The primary findings are illustrated as follows, (1) the novel strain parameters, including GLS, PSS and PSI obtained from the AFI algorithm can early identify myocardial dysfunction and predict adverse events in stable CAD with normal LVEF; (2) PSI and the number of LV walls with PSS are independent predictors of the endpoint; (3) the cutoff of PSI in predicting the adverse events is 10.4% with sensitivity 63.3% and specificity 81.0%; (4) for patients with normal or mildly reduced GLS, the prognostic value of PSS may be superior to GLS.

Coronary artery disease (CAD) is a pathological process characterized by atherosclerotic plaque accumulation in the epicardial arteries. The disease can have long, stable periods but can also become unstable at any time, leading to major adverse cardiovascular events such as myocardial infarction (MI), stroke and death [23]. Therefore, event risk assessment is an important step during the work-up for the patients with stable CAD since it has a major impact on the subsequent therapeutic decisions.

STE is recommended for the early diagnosis and risk stratification in a variety of cardiovascular diseases in recent years [5, 24]. Compared to the traditional echocardiographic parameters, it could provide more sensitive parameters in early identifying subtle myocardial damage, particularly contributing to improve the prognostic assessment in the patients with preserved LVEF [25, 26]. In the present study, speckle tracking AFI was applied in the routine work-up for patients with stable CAD to provide multiple deformation parameters fast and accurately. Consistent with previous studies, our study confirmed that both GLS and PSS obtained from the AFI algorithm could predict adverse events in stable CAD patients with normal LVEF. We found that increasing numbers of LV walls with PSS were related to impaired LV function. Differently, the predictive power of AFI parameters was further compared in the present study. Our results strongly supported PSS and PSI as independent predictors rather than GLS, indicating PSS may be more sensitive to pathological alterations and potentially ischemia than GLS [7]. In fact, the superiority of PSS to systolic longitudinal strain has also been indicated in other clinical scenarios. Voigt et al. [27] evaluated strain parameters during dobutamine stress in 44 patients with known and suspected coronary artery disease and compared the diagnostic accuracies of the deformation parameters with that of perfusion scintigraphy. PSS was observed during stress in all ischemic segments, and PSS parameters such as the PSI were better for identifying stress-induced ischemia than maximal strain in systole. It has recently been found that PSS may persist after brief ischemia even when peak systolic strain is recovered [14, 28]. For this reason, PSS is expected to be a valuable indicator and recognize the myocardial ischemic insults accurately. Brainin et al. [29] explored the diagnostic and prognostic potential of PSS in 293 patients with suspected stable angina pectoris. During a median follow-up of 3.5 years, a total of 25 patients (8.5%) experienced major adverse cardiovascular events. Although the presence of PSS remains an independent predictor of adverse events when GLS was added to the adjusted model, no significant difference in the prognostic performance for the primary end point was found between GLS and PSS. By contrast, the superiority of PSI to GLS in predicting adverse events was found in patients diagnosed with stable CAD with the absolute value of GLS > 15.4% in the present study (AUC = 0.73 [PSI] vs. 0.58 [GLS], *p* = 0.024). In Cox regression analysis, both PSS and PSI are independent predictors rather than GLS. It is reported that absolute GLS < 12% represents severe systolic dysfunction and adverse prognosis, and < 15%-16% seems to represent risk in patients with relatively preserved EF [30, 31]. Specifically, our results suggested that PSS may be a sensitive indicator to pathological alterations when strain in systole was preserved. PSS appears to provide prognostic information when GLS is normal or near normal. Our conclusion could be supported by another prospective study [32], where PSS provided prognostic information in general population with strain within normal range (GLS, − 17.1% ± 2.9%).

PSS was initially considered as a delayed but active contraction induced by ischemia [33]. However, many studies suggested that the contraction of the ischemic myocardium is affected not only by inherent contractility but also by tension from the surrounding non-ischemic myocardium [10, 34]. In a mathematical model study by Claus et al. [9], PSS was explained as passive recoil caused by the interaction between the ischemic and surrounding myocardium. Although the exact mechanism of whether PSS is caused by active contraction or passive recoil remains unclear, several studies demonstrated the close association of PSS and the adverse outcomes in type 2 diabetes [35], acute coronary syndrome [36] and even in the general population [32]. In our study, patients with pathological PSS had a lower systolic function. More importantly, we found that patients with an increasing number of walls with PSS had lower GLS and more adverse events. The incidence of events increased incrementally with the increase of PSI. These findings may involve two implications. First, PSS characterizes dysfunctional myocardial segments due to ischemic burden in the myocardium, which often occurs in conjunction with the relative reduction of longitudinal myocardial strain during ejection [11, 13]. Second, it is reported that the extent of PSS detected by STE is consistent with the ischemic area and the damage degree [7]. Therefore, a high PSI or more pathological PSS involved chamber walls is directly related to the extent of impaired myocardial segments, indicating the worse outcome.

## Limitations

There are still some limitations in the present study. First, the current study provides prognostic information on speckle tracking AFI multiparameter in patients with stable CAD. However, we believe it cannot be widely used for risk stratification in real-world clinical practice because of the strict inclusion and exclusion criteria in our study. Despite this, our findings confirmed the prognostic value of PSS in stable CAD patients with preserved systolic function, contributing to the risk stratification in those with relatively low-risk population. Also due to sample size limitations, the study used a combined end point including death and cardiovascular events, which may mask the predictive power for different endpoint events. Second, there may be overfitting in our multivariate model due to a lower incidence of adverse events. However, this could be acceptable when controlling for confounders as opposed to building prediction models [37]. Third, some studies suggested to identify pathologic from physiological PSS by analyzing the dynamic process of PSS. However, PSS is regarded as present if PSI is > 20% in the present study according to the criteria proposed by Voigt et al. We did not expire the relationship between the dynamic process of PSS and the adverse events. Last, it has recently been reported that early systolic lengthening (ESL) is also useful to detect myocardial ischemia and may provide some information on prognosis. Integrated analysis of myocardial deformational patterns should be explored in future studies.

## Conclusion

Speckle tracking AFI could provide useful prognostic information in stable CAD patients with preserved LVEF by early identifying myocardial dysfunction with strain parameters. There is strong evidence of the prognostic value of PSS for predicting major adverse cardiovascular events. Increased PSI or a greater number of LV walls with PSS indicate an increased risk of adverse events, and the prognostic value of PSS may be superior to GLS in patients with normal or mildly reduced GLS.

## Supplementary Information

**Additional file 1.** **Table S1.** Inter- and intraobserver variability for automated functional imaging parameters. **Fig. S1.** Schematic drawing of measurement of post-systolic index (PSI). PSI was calculated as follows: [(peak negative strain in cardiac cycle-peak negative strain in systole)/ (peak negative strain in the cardiac cycle)] × 100. **Fig. S2.** Flowchart of study population. CAD: coronary artery disease; LVEF: left ventricular ejection fraction. **Fig. S3.** Receiver operating characteristic curves of multiple parameters for evaluating adverse events. AUC: area under the curve; E/A: mitral inflow peak early velocity/mitral inflow peak late velocity; E/e': mitral inflow peak early velocity/mitral annular peak early velocity; LVEF: left ventricular ejection fraction; PSI: post-systolic index; GLS: global longitudinal strain.

## Data Availability

The dataset used or analyzed during the current study are available from the corresponding author on reasonable request.

## Abbreviations

AFI: Automated functional imaging
AUC: Areas under the curve
CAD: Coronary artery disease
CI: Confidence interval
E/A: Mitral inflow peak early velocity/mitral inflow peak late velocity
E/e′: Mitral inflow peak early velocity/mitral annular peak early velocity
GLS: Global longitudinal strain
HR: Hazard ratio
ICC: Intraclass correlation coefficient
IQR: Interquartile range
IVS: Interventricular septum
LAD: Left anterior descending artery
LCX: Left circumflex artery
LM: Left main artery
LV: Left ventricle
LVEDD: LV end-diastolic dimension
LVEDV: LV end-diastolic volumes
LVEF: Left ventricular ejection fraction
LVESV: LV end-systolic volumes
LVMI: LV mass index
LVPW: LV posterior wall
MACEs: Major adverse cardiovascular events
MI: Myocardial infarction
NYHA: New York Heart Association
PSI: Post-systolic index
PSS: Post-systolic shortening
RCA: Right coronary artery
ROC: Receiver operator characteristic curve
ROI: Region of interest
STE: Speckle tracking echocardiography
WMSI: Wall motion score index
