# Supplementary material for: Post-systolic shortening is superior to global longitudinal strain in predicting adverse events in patients with stable coronary artery disease and preserved systolic function
Source: Insights Imaging. 2022 Mar 2;13:35. doi: 10.1186/s13244-022-01174-y (PMC8891405; doi:10.1186/s13244-022-01174-y)
Supplement: Supplementary file 1 — Additional file 1. Table S1. Inter- and intraobserver variability for automated functional imaging parameters. Fig. S1. Schematic drawing of measurement of post-systolic index (PSI). PSI was calculated as follows: [(peak negative strain in cardiac cycle-peak negative strain in systole)/ (peak negative strain in the cardiac cycle)] × 100. Fig. S2. Flowchart of study population. CAD: coronary artery disease; LVEF: left ventricular ejection fraction. Fig. S3. Receiver operating characteristic curves of multiple parameters for evaluating adverse events. AUC: area under the curve; E/A: mitral inflow peak early velocity/mitral inflow peak late velocity; E/e': mitral inflow peak early velocity/mitral annular peak early velocity; LVEF: left ventricular ejection fraction; PSI: post-systolic index; GLS: global longitudinal strain. [file 13244_2022_1174_MOESM1_ESM.docx]

**ELECTRONIC SUPPLEMENTARY MATERIAL**

**Supplementary Table 1**

Inter- and intra-observer variability for automated functional imaging parameters

|  | Inter-observer variability | | |  | Intra-observer variability | | |
| --- | --- | --- | --- | --- | --- | --- | --- |
|  | ICC | 95%CI | P-value |  | ICC | 95%CI | P-value |
| PSI, % | 0.946 | 0.889-0.973 | 0.001 |  | 0.958 | 0.923-0.974 | 0.001 |
| GLS, -% | 0.935 | 0.903-0.960 | 0.001 |  | 0.943 | 0.932-0.987 | 0.001 |
| Walls with PSS | 0.957 | 0.939-0.971 | 0.001 |  | 0.971 | 0.967-0.988 | 0.001 |

ICC: Intra-class correlation coefficient; CI: confidence interval; PSI: post-systolic index; GLS: global longitudinal strain; PSS: post-systolic shortening.


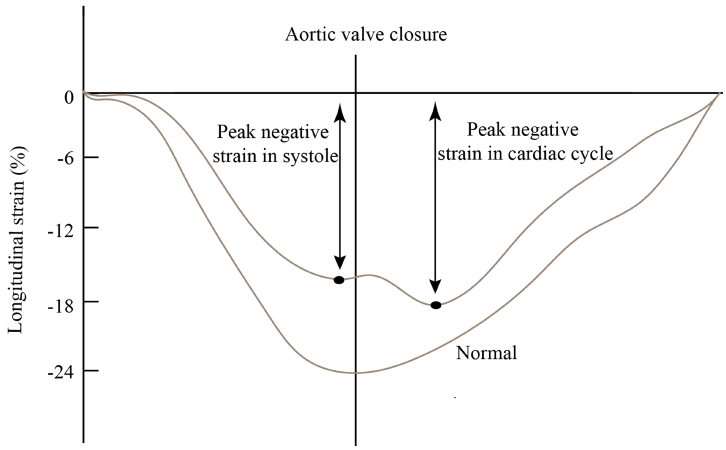


**Supplementary Fig. S1** Schematic drawing of measurement of post-systolic index (PSI). PSI was calculated as follows: [(peak negative strain in cardiac cycle-peak negative strain in systole)/ (peak negative strain in the cardiac cycle)] x100.


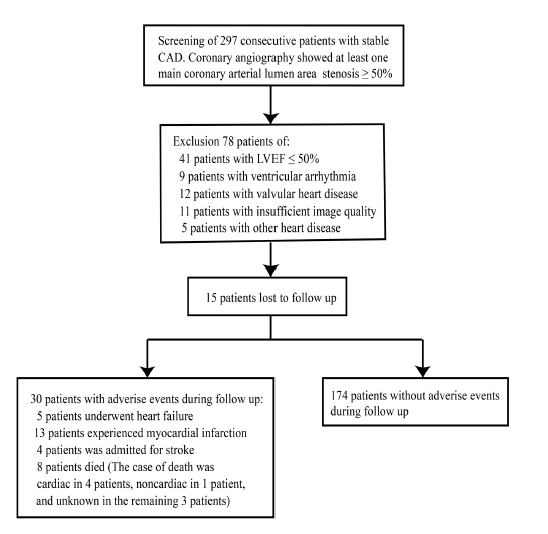

**Supplementary Fig. S2** Flowchart of study population. CAD: coronary artery disease; LVEF: left ventricular ejection fraction.


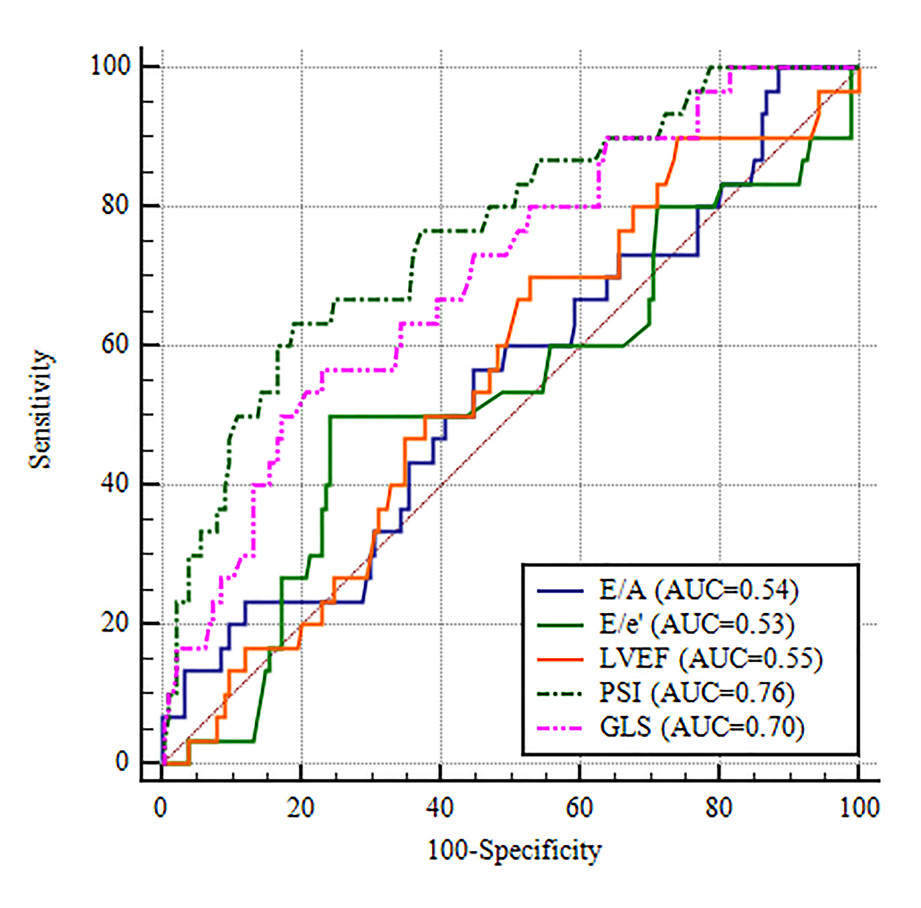


**Supplementary Fig. S3** Receiver operating characteristic curves of multiple parameters for evaluating adverse events. AUC: area under the curve; E/A: mitral inflow peak early velocity/mitral inflow peak late velocity; E/e': mitral inflow peak early velocity/mitral annular peak early velocity; LVEF: left ventricular ejection fraction; PSI: post-systolic index; GLS: global longitudinal strain.
